# Filling in the Gaps. Making Sense of Living with Temporomandibular Disorders: A Reflexive Thematic Analysis

**DOI:** 10.1177/23800844231216652

**Published:** 2024-01-03

**Authors:** C. Penlington, J. Durham, N. O’Brien, R. Green

**Affiliations:** 1 Newcastle University Faculty of Medical Sciences, Newcastle, UK; 2 Northumbria University Department of Psychology, Newcastle upon Tyne, Tyne and Wear, UK

**Keywords:** temporomandibular joint disorders, qualitative research, chronic pain, pain, chronic, facial pain

## Abstract

**Introduction:**

Persistent, painful temporomandibular disorders (TMDs) are challenging to manage and usually require the active engagement of patients. To achieve this, it is necessary to understand the complex and multifactorial nature of persistent pain. Many dental professionals have little education about persistent pain and may prefer to offer structural management and advice. This research aims to explore how people understand their persistent TMD and how this understanding has been influenced by their treatment providers.

**Methods:**

Twenty-one people were recruited to represent a diversity of experience with persistent TMD. Interviews followed a semistructured topic guide. Themes were constructed through reflexive thematic analysis to represent how people made sense of their symptoms and the messages that they had picked up through their treatment journey.

**Results:**

Participants described examples of conflicting opinions and inconsistent management recommendations. They rarely recalled collaborative discussions about the nature and complexity of their symptoms and different options for treatment. This experience is represented by a single theme, “a medical merry-go-round.” Subthemes of “a medical journey to nowhere—participants’ frustrated attempts to find medical management that will end their pain” and “is it me?—participants’ questioning their role in persisting pain” kept participants on the merry-go-round, while symptom resolution and participants’ emerging development of a holistic understanding of their TMD pain provided exit points. Understanding pain holistically tended to be helpful and typically occurred despite rather than because of the advice given in routine treatment settings.

**Conclusion:**

Participants in this study had not typically found their pain management within dental and medical settings to have helped them to construct meaning and understand their experiences of painful TMD. However, understanding symptoms holistically was experienced as beneficial. This study suggests that improved communication and signposting within services for persistent TMD may be beneficial to patients with TMD pain.

**Knowledge Transfer Statement:**

Results of this study confirm that being offered a series of anatomically based, singular-cause explanations for persisting pain symptoms had been experienced as unhelpful by the participants who had sought help for their TMD. Participants highlighted the importance of accurate and collaborative communication and of dental professionals explicitly adopting and communicating a biopsychosocial understanding of pain to their patients who have TMD. Results highlight that some people can struggle to manage persisting pain with minimal support. Signposting patients to appropriate services and resources may help them to understand more about the nature of persistent pain and methods of managing it.

## Introduction

Temporomandibular disorders (TMDs) represent a group of disorders that are frequently painful and can limit jaw function (Greene 2010). Prognosis can vary; although symptoms frequently resolve, up to 49% of people with painful TMD continue to experience symptoms 6 mo later (Slade et al. 2013). The International Association for the Study of Pain (IASP) defines chronic pain (referred from here onward as persistent) as pain of at least 3-mo duration. Management is challenging in many cases (Durham et al. 2007) and accompanied by a high level of uncertainty (Garro 1994; Durham et al. 2010).

Helpful and supportive conversations about pain between patient and dentist (or another clinician) are important and can be challenging to get right. In part, this may be because comprehensive pain education is lacking in health care curricula, including medical and dental training (Thompson et al. 2018; Costa et al. 2021). Related skills in communication, critical appraisal, and working with interprofessional colleagues are also important but missing from typical dental education (Costa et al. 2021). Advances in how pain is understood (Suvinen et al. 2005; Penlington et al. 2019) have increased complexities in working with and communicating about pain. Much persistent pain is now recognized to be neuroplastic in origin (Moseley and Butler 2015). Neuroplastic pain refers to pain that is caused by the functioning of the pain system itself rather than structural factors within the body. Despite decades of scientific understanding indicating that TMD, like other conditions, is best understood within a biopsychosocial framework (Dworkin 1994), unidimensional views remain prevalent in both medical/dental and cultural settings in Western countries (Gross et al. 2006; Bonathan et al. 2014; Darlow et al. 2014).

If pain is understood as multifactorial, then it makes sense to consider the “whole person” in any assessment, asking questions about lifestyle impact and mood and including such dimensions in a management plan. However, doing so makes less sense and may be misconstrued as dismissive if pain is considered primarily biomechanical in nature by either patient or clinician. Dentists are highly familiar with considering structural causes of pain, both through their education and routine practice. This way of understanding pain may limit their ability to understand and explain persistent pain conditions such as TMD. For example, in 1 study, dentists expressed a strong preference for treating TMD with oral splints over other treatment options (Aggarwal et al. 2012). It is feasible that this preference for a simple anatomical form of management may impact the experience of patients who have symptoms not easily managed by this method.

As with other chronic pain conditions, a good outcome for TMD might not always involve relief of pain (Hush et al. 2009); quality of life and ability to function can be seen as equally important. Whether the primary goal of management is oriented predominantly to reduce or to live well with pain, the “right thing to do” will depend on the particular views held by people with TMD and clinicians about the nature of the problem and range of acceptable outcomes. Beliefs about whether pain has been validated or taken seriously are similarly likely to depend on these views.

The aim of this research was to explore how people with persistent TMD make sense of their pain and how the messages that they have picked up through their health care journey may have influenced their understanding and experience.

## Methods

Qualitative approaches to research explicitly recognize that research is not value free but is shaped by an individual according to their (explicit or implicit) assumptions about the world and the nature of knowledge (Creswell 2007). It is important therefore to be explicit and aware about these assumptions and how they may influence the research process. This is often done by positioning research (and researcher) within a philosophical framework, which takes a position on the nature of reality (ontology) and of what can be known (epistemology). The framework adopted for this research is critical realism. Critical realism (Bhaskar 2020) is able to embrace a constructivist epistemology that acknowledges that individual experience varies due to many factors and that no 2 people will experience the same events in the same way, while also embracing a realist ontology that recognizes that such experiences are underpinned by real events and have real causes that may or may not be observed (Fletcher 2017).

This research followed a broadly experiential design, aiming to document participants’ experiences in clinical settings and the meaning-making about their pain that came from this. The method used was reflexive thematic analysis, within which a critical realist approach (Bhaskar 2020) was adopted. Reflexivity is an important part of qualitative research and involves the researcher remaining aware of their position and how it influences their assumptions and choices on an ongoing basis (Clarke and Braun 2018).

### Reflexive Statement

I (the first author and person who conducted the interviews) am a clinical psychologist with several years’ experience working in pain management settings. During this time, I have been privileged to share many accounts of people living with persistent pain and help them to develop new and more effective ways of relating and responding to their pain experience. I am familiar with current theories of pain science and approaches to pain management and with service pathways in my own country, England. This includes an awareness of the limitations of current provision for persistent pain and a wish to improve it.

### Ethics

Ethical approval for the research was granted by the GMWest NHS ethics review panel, ref. 20/NW/0155. Following initial contact and screening, participants read an information sheet about the study, filled in a written consent form, and selected a convenient date for an online interview. Interviews were recorded and transcribed. Transcripts were checked for accuracy by the first author and then stored on a secure server with identifying information removed. A key was stored separately to enable data to be removed if requested by a participant.

### Sampling Strategy and Rationale

A criterion-based, maximum variation, purposive sample was planned to obtain a depth and breadth of experiences across the demographics and presentations of TMD and to allowed for exploring potentially differing experiences. The criteria were gender, ethnic group, age, duration of pain, and presence of comorbidities. To meet this aim, a range of recruitment methods was used. Eligible participants were initially recruited from a secondary care dental setting in Northeast England, where they were approached by clinical staff and asked if they would consent to receive information from the researcher. Since this was the primary recruitment method, most of the participants interviewed had attended an appointment with a specialist. For participants recruited through these clinics, clinical diagnosis was confirmed by clinical staff. Study posters were also displayed within the same secondary care setting and surrounding areas. As the study progressed, eligible patients of the clinic who had previously consented to receive information about research studies were contacted if they were part of a demographic yet to be sampled for the study (in this case specifically, male and/or from an ethnic minority). Additional recruitment, again targeted toward including undersampled groups, was conducted via social media and through 2 support organizations for people living with persistent pain, the Temporomandibular Joint Association (TMJA) and Footsteps Festival, an online site that runs free events to support people with persistent pain. Participants who were not recruited through a clinical setting were asked to fill in a screening questionnaire consisting of 6 questions about the presence or exacerbation of jaw pain within the past 30 d (Gonzalez et al. 2011) to confirm eligibility—a score of 3 or more was used to indicate the probable diagnosis of TMD.

Sample size was determined by Information Power (Malterud et al. 2016) based on a rubric considering the study aim, sample specificity, use of established theory, quality of dialogue, and analysis strategy. Information Power provides a meaningful alternative to the concept of data saturation, which is both inextricably linked to specific analytical strategies (Braun and Clarke 2021) and is also theoretically unlikely (O’Reilly and Parker 2012) given the expectation that all relevant data have been sampled. Information Power provides a guide for determining when enough information has been gathered to offer new insights or challenge existing ideas on a topic (Malterud et al. 2016). Appendix Table 1 describes how the rubric was applied to determining sample size in this study. The final sample included 21 participants, of whom 8 were male, 7 from an ethnic minority, ranging in age from 19 to 72 y and with symptom duration from 3 mo to 32 y. Demographics of the sample are shown in the Table.

**Table.** Demographic Characteristics of Study Participants.

Participant	Pseudonym	Gender	Age Range	Ethnic Minority?	Symptom Duration
1	M	Female	26–40	N	1 y
2	Z	Female	18–25	N	6 mo
3	J	Female	26–40	N	2 y
4	X	Female	41–60	N	1 y
5	F	Male	26–40	N	1 y
6	K	Female	18–25	N	5 y
7	H	Female	26–40	N	12 y
8	G	Male	18–25	N	1 y
9	L	Female	26–40	Y	8 mo
10	D	Male	18–25	Y	3 mo
11	I	Male	26–40	Y	4 mo
12	P	Male	26–40	Y	1 y
13	Y	Female	41–60	N	25 y
14	B	Female	41–60	N	30 y
15	W	Male	18–25	N	9 mo
16	V	Female	41–60	N	32 y
17	T	Female	61+	N	25 y
18	C	Female	26–40	Y	2 y
19	R	Female	61+	Y	23 y
20	E	Male	61+	N	30 y
21	A	Male	41–60	Y	10 y

Table shows pseudonym allocated to each study participant along with their gender, age range, ethnic minority status, and duration of symptoms.

### Process

Participants were interviewed online using a semistructured topic guide developed by discussion between all authors. All interviews were carried out by the first author between September 2021 and October 2022. The topic guide was followed flexibly and reviewed after each interview and modified to further explore some of the experiences brought up by participants. Interviews were recorded and transcribed verbatim by a professional transcriptionist. Interviews lasted from 24 to 108 min with a mean of 58 min. The first author checked the accuracy of transcriptions and replaced names of participants with single-letter pseudonyms to preserve anonymity. At this stage, recordings were deleted and anonymized transcripts stored on a secure server accessible only to the study team.

Reflexive thematic analysis (Braun and Clarke 2006) was followed. Codes were assigned by the first author (C.P.) to each interview in turn, taking a semantic approach in order to capture as closely as possible what was actually being said. In keeping with a critical realist orientation, participants were assumed to be capable of accurately expressing their own experience and meanings, so coding was carried out descriptively keeping close to the meaning expressed in the texts. Codes were assigned using double columns on Microsoft Word and later grouped into larger units with similar meaning. The first author independently grouped units into candidate subthemes and themes that were discussed between all authors. At this point, the procedure became more analytical, considering information excerpts in the context of the additional knowledge and perspectives of the authors about pain, from clinical and personal experience and from the literature. Three authors (C.P., R.G., J.D.) met to collaboratively regroup the subthemes to highlight differences in perspective that might shed light on unexpressed assumptions. Overall differences were resolved by consensus discussion and slight adjustments made to the candidate themes. In addition to this discussion, one of the supervisors (R.G.) carried out a detailed audit of the first theme identified, checking each subtheme back to its constituent codes and quotes.

## Results

This article reports results regarding the journey experienced by participants through treatment and toward their current understanding and management of their painful TMD. All participants had a clinically confirmed or screening questionnaire-based diagnosis of TMD with or without comorbidities, which included migraine, fibromyalgia, Sjögrens disease, and chronic fatigue syndrome.

An overarching theme of “a medical merry-go-round” was constructed by the authors to capture key narratives about the journey so far. The image of a merry-go-round was felt to capture the cyclical and repeated experience of seeking, being recommended, and trying the next treatment modality, often without a full explanation of the rationale and without benefit. Included in this merry-go-round are subthemes of “a medically focused journey to nowhere—participants’ frustrated attempts to find medical management that will end their pain” and participants questioning their own role in persisting pain symptoms, “is it me?” Exit from the roundabout might occur by “participants’ emerging development of a holistic understanding of their TMD pain,” which is a third subtheme. Another way of leaving the merry-to-round would be the resolution of symptoms. Such resolution was not the focus of the current research and is not a subtheme but rather is mentioned as an acknowledgment that symptoms do sometimes resolve and provide an exit from the cycle of continual treatment and sense-seeking for people with TMD.

A thematic map of the results is represented in the Figure.

**Figure.**
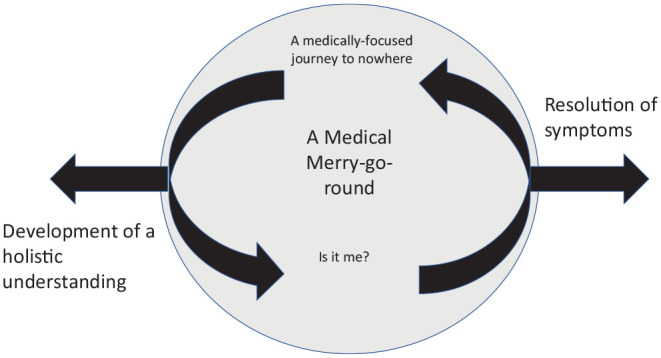
Thematic map representing findings about the treatment journey of participants’ experiencing persistent temporomandibular disorders.

### A Medically Focused Journey to Nowhere—Participants’ Frustrated Attempts to Find Medical Management That Will End Their Pain

Those interviewed described going to see doctors or dentists looking for answers regarding their pain. Participants described multiple consultations that all too often left them grappling with mixed messages and explanations that did not fit their experience, recalling varied accounts of the information that had been given to support management. The initial message that J was offered by her dentist was extremely negative, whereas M was offered almost nothing in the form of explanation.

And then very soon, I went to see my dentist. And he said straight away, there is no question in my mind, this is TMD. . . . He looked visibly upset about it, he said I really hate telling people that they have that because there’s nothing can be done about it, it isn’t going to go away. And at the time I was just, I was struggling at that time to even function. (J, female, age 25–40, symptoms for 2 y)

Then [the dentist] just disappeared out the room, so I was just sat there with the dental nurse thinking what’s happening now, and then she just came back with some bits of paper and she was like “Oh there you go, you can read up on, you know what more you need to do. And it was a couple of exercises to do. . . . She never explained anything about it. (F, male, age 25–40, symptoms for 1 y)

Participants’ accounts suggested typical management based on serial singular (sometimes implied) explanations and solutions for their pain, which could be contested between their health care providers, as illustrated by the following quotes:

My dentists kind of gave me jaw exercises and then I went to the dentist they told me to and they said that they were the worst thing I could be doing for my jaw. (K, female, age 18–25, symptoms for 5 y)

They went through all of the horrible tablets that they have you on and all these they’re supposed to . . . they don’t take any pain away and they just make me feel sick. (H, female, age 26–40, symptoms for 12 y)

The combination of serial unsuccessful treatments with limited explanation and communication about the condition could leave participants uncertain about what was happening with their condition and how it should be managed. This sense of uncertainty was shared by the majority of participants, but notably not by 2 who had professional experience themselves of working within dental settings. Despite ongoing problems with pain and, in 1 case, closed locking of the jaw, each of these individuals expressed a confidence and acceptance of their symptoms that differed from many of the other participants.

I accepted it fairly well because I knew from my background that was the sort of thing that I would be told and that it’s not something, unless it’s very bad, it’s not something you would have regular dental visits about. (B, female, age 41–60, symptoms for 30 y)

It helped because I worked there [dental setting] as well so it wasn’t seen as just let’s have an appointment and chat about it, I could pick his brain every now and then rather than a sit down and a full chat. (V, female, age 41–60, symptoms for 32 y)

Most participants, however, did not have the benefit of prior knowledge about the nature and management of TMD. Many had experienced communication within dental or medical settings that seemed to range from simply unhelpful to potentially detrimental. To improve communication, M (female, 26–40, symptoms for 1 y) suggested that “a checklist of what they are looking for, and a diagram” could be of benefit, and H (female, 26–40, symptoms for 12 y) felt that when assessing for parafunctional oral habits that she may not immediately be aware of, “if they’d said can you take a week to assess situations, to take notes if and when you are,” would have led to a more accurate conclusion. On a broader level, the impact of a lack of communication that was focused on the whole person within consultations where participants were desperate for help could be profound. For some participants, the conversation about managing persistent pain had never occurred, despite many consultations regarding the condition including at specialist level.

So receiving news of no positive outcome or management I think I would have felt happier, not happier, happier is not the right word. I think I would have felt more positive if he had said to me well that medically there’s nothing we can do however, to help you cope mentally try these techniques. (C, female, age 61+, symptoms for 23 y)

Overall, participants had frequently consulted medical and dental practitioners looking for answers about their pain as well as a solution but were often frustrated in both aims. The combination of serial simple management options that had not helped, along with limited opportunities to discuss persistent pain or more holistic understandings of pain, could leave participants with gaps in understanding their symptoms and reasons that they were not resolving.

Where participants’ symptoms had not resolved and management had not helped them to understand the nature of their condition, they turned to their own explanations and potential solutions. For some, this led them to question the validity of their own experiences and ways of responding.

### Is It Me?—Participants Questioning Their Role in Persisting Pain Symptoms

A culture where pain is not clearly understood and not always taken seriously, whether in clinical settings or closer to home in a social context, can affect how people with pain evaluate themselves. A situation where management has been recommended and is expected to lead to an improvement but where there is no improvement will inevitably cause dissonance. This dissonance is expressed by Y in relation to recommended jaw exercises.

You’re trying exercises and your jaw’s got really tight and it’s all about tension and when I tried to do the exercises that they were triggering it even more. (Y, female, age 41–60, symptoms for 25 y)

Resolving dissonance is important. One way of doing so might involve rejecting the explanation and advice that has been offered regarding management of symptoms; however, doing so risks further uncertainty and potential loss of hope if there is no immediately accessible plausible management option. Despite the effort taken to maintain a positive attitude, X explains that doubts can creep in about her own role in perpetuating an ongoing struggle with her symptoms.

It’s almost thinking, if I’m being very very honest it’s almost like I’m thinking I’m failing, like I’m not doing, well I am following the instructions I’ve been given and trying to do things and being careful, but am I failing what I should be trying to do? . . . am I just being daft really and not thinking properly well I’m I know I am thinking, properly, but sometimes I think no. (X, female, 41–60, symptoms for 1 y)

In attributing her lack of progress to a potential shortcoming or lack of effort or skill on her part, she is able to avoid falling foul of the medical system and thus being left without hope of a resolution. Taking responsibility for perhaps not getting things right or not trying hard enough also allows the illusion of control, as things may improve were she to do better. There is a high price to pay for retaining this safety net, though—her own sense of agency and competence.

Persistent pain may be poorly understood in both medical settings and the community. Participants talked of friends or family who minimized their experience of pain. Characteristics of pain, including its variability, invisibility, and longevity, go against common perceptions of illness and pain and could lead to individuals being disbelieved and blamed by family or friends—especially at times of low-mood participants, who themselves may also share such culturally held conceptions of pain, could question their own experience.

If it’s a bad day I can get really down on it and sometimes I think it’s not real. Obviously, it is, I have all of these diagnoses and I’m on all of these meds but sometimes I’m like are you just exaggerating? (W, male, 18–25, symptoms for 9 mo)

This tendency of some people to question themselves when their experiences do not fit with what they think or are told they should be experiencing is important as it may cause additional uncertainty and distress and interrupt their ability to successfully manage their pain. In this study, it presented as 1 of 2 common responses to the experience of pain that does not fit with the received (but incorrect) wisdom that real pain is associated with observable pathology and will predictably respond to simple management. The alternative response is to question how pain is conceptualized and perhaps come to a more holistic understanding.

### Participants’ Emerging Development of a Holistic Understanding of Their TMD Pain

Where some participants had developed a broader and more holistic understanding of their symptoms, this understanding was rarely a result of the consultations that they had attended. Rather, it may have been introduced to them by friends or as a result of their own reflections or research. When medical advice did not help, R described seeking answers from friends and family:

When I discovered that I have this chronic pain, that I found maxo facial, the doctors, they weren’t really apart from offering me loads of drugs, they weren’t really helping. . . . So I researched and looked into it myself and spoke to friends and they said try this, try that. (R, female, 61+, symptoms for 23 y)

The offer of primarily physical interventions aimed at perceived biomedical problems with anatomical structures suggests a view that such options are most important in managing pain. Such a view is inconsistent with the biopsychosocial model, which has been recognized in TMD management for some time. Despite this, participants did not tend to recall any routine acknowledgment of personal and contextual factors in their consultations. It may be that some dental and medical practitioners do not consider their role to include providing education and support around the psychosocial elements of pain. This is illustrated by a quote from C.

I’ve noticed like that binary, there was like, you know, that there’s . . . the way we look in like the Western world that these two things are separate but dealing with the TMJ it’s like oh this is a body thing, but it’s deeply connected to how my mind is feeling at the moment. So I’ve had to learn to navigate that better. . . . I think part of the issue is that by that point you may already be reaching out to professionals who actually can’t help you. Like a dentist who’s already said oh yeah, like well your problem is you grind your teeth so we solve it by giving you this mouth guard and then you walk away and it’s like OK that’s all I need to do, but maybe you need like a big picture. (C, female, 26–40, symptoms for 2 y)

In the absence of integrated biopsychosocial management within routine medical and dental care, participants were left to construct their own ways of integrating medical and psychosocial constructs. Experience of anxiety and of being a mental health first aider helped F to recognize the value of taking such a holistic approach to his physical symptoms.

I’m a mental health first aider as well with my anxiety issues. . . . These are the kinds of things you can suggest . . . and sometimes you do need to sort of apply them to yourself. (F, male, 26–40, symptoms for 1 y)

Although it may be helpful, understanding pain holistically can be more complex than relying on the resolution of a structural problem. The result is that even with relevant knowledge, it can be difficult to assimilate behavioral explanations if expecting a structural or medical solution. This is reflected by J, who was told by a specialist after a year of intense pain that her jaw was essentially normal, and she needed to relax. Her initial reaction was anger:

And then also it kind of felt like to me as well, I’d waited so long and it just felt like an end, then there was like this examination and she just said, like, there is nothing wrong with you. (J, female, 26–40, symptoms for 2 y)

On reflection, J was able to assimilate reassurance that she was given and advice to relax her jaw with previous experience and knowledge, gained through experiencing other painful conditions and through training in massage, to work toward a positive outcome. The reassurance that she was given at her TMD appointment was the key piece of information that she needed to activate her existing skills in self-management. She explained how the consultation she attended might have more seamlessly led her toward this awareness of what she needed to do.

I think for me I needed more kind of emphasis on the—this is why this thing is going to fix this thing. If you do this, it will help with this. Like, more of a, in-depth, chain effect, kind of thing.

Overall, reaching a holistic understanding of symptoms was not easy, but for those who had done so, all had found it to be helpful. This was not the case for participants who continued to hold more targeted structural explanations for their symptoms and to experience pain. Universally, understanding symptoms in a broader and more holistic way included elements outside of clinical consultations. Frequently, rather than acknowledging and signposting resources for managing the impact of symptoms on the whole person, dental and medical consultations presented barriers to holistic understanding and maintained a focus on simpler and more structurally based treatment modalities.

## Discussion

The central theme constructed within this research was “a medical merry-go-round.” The theme was developed to capture the essence of participants’ accounts of going round in circles but often not moving forward in the quest to find answers and solutions to their pain. The theme was developed further with subthemes of “a medically focused journey to nowhere—participants’ frustrated attempts to find medical management that will end their pain,” “Is it me?—participants questioning their role in persistent pain symptoms,” and “participants emerging development of a holistic understanding of their pain problem.”

The pattern of cycling between unsuccessfully seeking medical or similar solutions and questioning or doubting oneself when such solutions are unsuccessful reflects similar literature published over the past 30 y (Garro 1994; Durham et al. 2010; Taimeh et al. 2023a). One study described participants as occupying a liminal space between healthy and sick as they struggled with symptoms and questioned their own legitimacy (Durham et al. 2010) prior to diagnosis. In the current research, this uncertainty could persist beyond diagnosis if symptoms persisted. Recent research from a different country (Sweden) similarly highlighted the experience of TMD patients not receiving treatment within dental settings that fully meets their needs (Ilgunas et al. 2023). Despite significant advances in the field since the introduction of the biopsychosocial model to the management of TMD (Dworkin 1994; Penlington et al. 2019), it appears that little may have changed in the experience of some people seeking care. A preference for medical solutions and reluctance to consider the relevance of more personal factors was linked to a philosophy of mind–body dualism (Garro 1994) that continued to be present in the descriptions of current participants regarding the care that they had received and, for many, their own views of their condition. However, this may have been partly the result of the sampling strategy, which relied heavily on recruitment through secondary care clinics. A recent study that recruited a largely community sample (Eaves et al. 2015) reported that the majority of participants accepted their ongoing symptoms of TMD without multiple consultations or seeking ongoing care.

It appears that continuing to seek medically based answers beyond a certain amount of unhelpful treatment is commonplace but may be counterproductive. Due to the mismatch of people’s continued experience of pain with what they and others expect, seeking additional answers and validation from services that cannot help appears to put them at risk of invalidation and self-doubt. This insight echoes similar findings from related research (Garro 1994; Durham et al. 2010; Ilgunas et al. 2023; Taimeh et al. 2023b), reflecting consistency over a prolonged period of time, during which it might have been expected that advances in understanding the multifactorial nature of pain (Moseley and Butler 2015) would lead to changes in patient experience. A question that remains is, why do some people persist in seeking answers based on the position or health of the tissues to their painful TMD in the face of serial treatment disappointments when others move on to developing a different way of understanding, managing, and relating to their pain?

One reason may be based on the beliefs about pain, of both participants and their dentists. In Western cultures, the belief that pain is a structural issue remains strong, and much health care training continues to focus on structural elements of pain (Thompson et al. 2018). Consultations may be based on, or convey, a dualistic model that implicitly communicates that psychological factors mean that pain is “psychosomatic” or “in the head” (Wolf et al. 2006). The strong possibility that either dentist or patient will hold such a dualistic model of pain suggests a need for careful and tailored conversations within consultations. As reported previously (Bonathan et al. 2014), the lack of such conversations could lead to differences between what dentists and their patients believed to have been discussed and understood in a consultation. From the perspective of participants in this study, at least, detailed conversations about the nature of pain and importance of holistic factors had not been a part of their experience in dental or medical care.

A small number of participants in this study did develop a holistic understanding of their symptoms, although this appeared to be despite, rather than because of, their medical and dental care journey. Doing so led to them being able to exit the “merry-go-round” and sometimes even to find something positive in their experience, such as a lesson to live life differently. These findings are similar to those reported many years ago (Garro 1994), with 2 study participants reported as taking positive meaning from their ongoing symptoms.

Within the current study, participants often expressed dissatisfaction about how their diagnosis had been conceptualized and communicated. Some gave examples of doctors or dentists who reportedly did not listen. In other instances, the issue may rather have illustrated difficulties around communicating about pain given differing initial views about what pain is. The lack of a standardized diagnostic pathway, damaging effects of uncertainty, multiple treatment episodes, and mixed messages reported in this study are also consistent with existing research about experiences with TMD (Garro 1994; Durham et al. 2010; Bonathan et al. 2014). In addition to the uncertainty described in our study, some participants, who continued to follow prescribed advice but saw little improvement in their symptoms, reported self-doubt or blame, wondering if their lack of improvement was the result of not trying hard enough or even whether they were making the pain up. The importance of validation and the tendency to both experience invalidation when seeking answers to pain and question the validity of one’s own experience of pain is also reflective of previous research on the topic (Garro 1994; Bonathan et al. 2014).

Some of the changes suggested in this research could easily be adopted clinically, such as the suggestion made by 1 participant to show patients information about what checks are being carried out during investigations. This may have the effect of supporting patients with the uncertainty of their condition by providing a framework of clarity around what is and is not known. It would seem important also to provide well-targeted information at an early stage about the multifactorial nature of pain. Routine and sensitively delivered information about the complex and multifactorial nature of pain might have the effect of changing the perceived social context to one of threat (if messages are interpreted as personally stigmatizing or attacking) from support. However, this may be beyond the communication skillset of many dentists currently. Further research should explore the impact of dentists’ communication skills on the management of persistent TMD and whether this can be improved with targeted education about the complexity of pain and about communicating this complexity.

Another useful avenue of future research may be to examine the characteristics of people who are able to develop a more complex and holistic understanding of their pain. It seems that reducing or managing uncertainty may be one important factor in this, as the 2 participants with dental backgrounds who had low levels of uncertainty about their condition were less concerned and better able to accommodate symptoms. The concept of intolerance of uncertainty (Carleton 2012) would be interesting to explore as a potential factor that could shape both the communication from clinicians (Simmonds et al. 2012) and pain experience of patients (Zaman et al. 2021). Similarly, interventions that have helped patients to develop psychological flexibility (McCracken and Morley 2014) have shown success in helping people to live well with pain. Further research could explore whether psychological flexibility is associated with successful management of TMD and whether it can be enhanced by certain messages during the journey through care.

There are some limitations to this research. Despite efforts to recruit a sample that had a depth and breadth of views, the predominant sociodemographic of the sample were White females. Having said that, the sample is in keeping with the gender distribution commonly seen in TMD (Bueno et al. 2018). Targeted recruitment to groups underrepresented in early interviews included recruitment through social media channels, which generated a more diverse response. However, for these (4) participants, we were relying solely on self-report of their symptoms. Internet connection, language barriers, and, for 2 participants, a lack of detail to their responses caused these responses to be less rich and trustworthy than other interviews, which was an unintended consequence of a social media recruitment method intended to improve diversity. The recruitment from a wider geographical pool, coupled with national restrictions in the light of the COVID-19 pandemic, also meant that interviews needed to be conducted online. Disadvantages of this approach might have been that it presented barriers to participation for people without access to or familiarity with the relevant technology (Lobe et al. 2022), typically older, poorer, and more rurally based people. On the other hand, digital interviews may also have increased the accessibility of the study by removing the need to live locally or travel to a specific location (Lobe et al. 2022).

This difficulty raises the question about how qualitative interview studies and health research more generally can be designed to ensure that diverse participants are meaningfully engaged and involved. In the interview setting, language, social class, and cultural differences and sensitivities can affect how accessible and possible participation is (Mellor et al. 2014; Kerrison et al. 2023). Consideration of the gender, language, ethnicity, and class concordance between the participant and the interviewer is recommended (Mellor et al. 2014). It is important therefore to acknowledge that despite our efforts to recruit from a diverse range of backgrounds, there were limitations in this respect. In addition, most (although not all) participants were from England, which may have limited the findings and resulted in a majority of experiences being reported from the perspective of English health care.

Overall, however, the richness of the data and efforts to recruit participants from diverse backgrounds are strengths of the study. Data management practices, including prolonged engagement with the data and team discussions, were consistent with recommendations for credibility in qualitative research (Nowell et al. 2017). Confirmability is supported by an internal audit of analyses and supports the overall trustworthiness of findings (Nowell et al. 2017). The findings are consistent with both recent (Ilgunas et al. 2023) and previous qualitative research on the topic. The experience of patients seeking answers and treatment for TMD was previously explored over 10 y ago (Garro 1994; Durham et al. 2010) when current understanding of pain was less well developed. This research answers the question of whether patients pick up more accurate and helpful messages about pain because of their treatment journey, which unfortunately was not the case for our participants. The research supports much of the previous qualitative literature about the experience of people with TMD and adds insights about the impact of understanding persistent pain within a structural or biomedical framework and the importance of prioritizing more biopsychosocial-based conversations about pain.

## Conclusion

Participants in this study reported limited explanations of their persisting TMD. The explanations reported either implicitly or explicitly prioritized structural, biomedical views of their persisting pain. Understanding pain in this way could keep participants trapped on a “merry-go-round” of treatment seeking and self-doubt, unless they were able to develop a broader and more holistic view of their symptoms.

## Author Contributions

C. Penlington, contributed to conception, design, data acquisition, analysis, and interpretation, drafted and critically revised the manuscript; J. Durham, R. Green, contributed to conception, design, data analysis and interpretation, critically revised the manuscript; N. O’Brien, contributed to design, data interpretation, critically revised the manuscript. All authors have their final approval and agree to be accountable for all aspects of work.

## Supplemental Material

sj-pdf-1-jct-10.1177_23800844231216652 – Supplemental material for Filling in the Gaps. Making Sense of Living with Temporomandibular Disorders: A Reflexive Thematic Analysis Supplemental material, sj-pdf-1-jct-10.1177_23800844231216652 for Filling in the Gaps. Making Sense of Living with Temporomandibular Disorders: A Reflexive Thematic Analysis by C. Penlington, J. Durham, N. O’Brien and R. Green in JDR Clinical & Translational Research

sj-pdf-2-jct-10.1177_23800844231216652 – Supplemental material for Filling in the Gaps. Making Sense of Living with Temporomandibular Disorders: A Reflexive Thematic Analysis Supplemental material, sj-pdf-2-jct-10.1177_23800844231216652 for Filling in the Gaps. Making Sense of Living with Temporomandibular Disorders: A Reflexive Thematic Analysis by C. Penlington, J. Durham, N. O’Brien and R. Green in JDR Clinical & Translational Research
