# Supplementary material for: Filling in the Gaps. Making Sense of Living with Temporomandibular Disorders: A Reflexive Thematic Analysis
Source: JDR Clin Trans Res. 2024 Jan 3;9(4):358–67. doi: 10.1177/23800844231216652 (PMC11408972; doi:10.1177/23800844231216652)
Supplement: sj-pdf-1-jct-10.1177_23800844231216652 – Supplemental material for Filling in the Gaps. Making Sense of Living with Temporomandibular Disorders: A Reflexive Thematic Analysis [file sj-pdf-1-jct-10.1177_23800844231216652.pdf]

Supplementary Materials. Filling the Gap study of patients experiences of seeking treatment for TMD; Penlington, C, Durham, J, O'Brien, N, Green, R.

Supplementary table 1

Illustration of how information power was applied to determine the final sample size of 21

Phase	Aim	Specificity	Theory	Dialogue	Analysis
Rubric	Broad study aim requires a larger sample than a narrow aim because phenomenon under study is more comprehensive.	High information power is achieved by recruiting participants with a specific index experience (TMD) and also some degree of variation within the experiences to be explored. A sample which includes those with experiences not previously described adds to information power.	A study supported by limited theoretical perspectives would usually require a larger sample to offer sufficient information power than a study that applies specific theories for planning and analysis.	A study with strong and clear communication between researcher and participants requires fewer participants to offer sufficient information power than a study with ambiguous or unfocused dialogues.	An exploratory cross-case analysis requires more participants to offer sufficient information power compared with a project heading for in-depth analysis of narratives or discourse details from a few, selected participants.
7 interviews recruited through local dental hospital consultant clinics.	All participants have painful TMD. For some this is just one of many pain conditions they have. The impact on them varies according to multiple factors and this affects their experiences and views	The study deliberately aims to recruit a broad sample in terms of age, gender, ethnicity, impact of pain, specific and co-morbid diagnoses – however at this stage 6 of 7 interviews have been white females and	The design of the study is not pre-determined by a specific theory. Aim is to take a phenomenological perspective and to ground data about factors influencing self-management in the lived	Researcher is an experienced, trained interviewer who is familiar with the condition and issues that impact people who have it. Semi-structured topic guide has been carefully developed and	Design is cross-case analysis. However, there is the aim for some depth of discussion in keeping with the aim of understanding in some depth the individual experiences of people

	of self-management. The aim of the study incorporates understanding these experiences therefore is relatively broad.	all recruited from the same dental hospital. There are variations represented in how people approach TMD and the impact it has.	experiences and beliefs / understanding of the people interviewed.	informed by existing knowledge of the condition.	who are living with TMD.
Information power	Low – more interviews needed as scope is broad.	Low – more variation needed both in terms of demographics and care received.	Low – more interviews needed as data collection A not overtly guided by theory.	High – good quality information included and most interviews are close to an hour in length.	Mixed. Larger number likely needed due to cross-case nature.
4 additional interviews recruited through general social media	Breadth of study aim is not affected by additional interviews	This recruitment method increased the diversity of the sample within the same target experience – self-management of TMD. Self-reported nature of the problem along with public dissemination strategy potentially introduced people with diverse motivations for engaging though and questions over whether all genuinely had TMD which are difficult to answer.	Not closely guided by theory.	Dialogue from these participants was much more sparse and of poor quality. Lots of background noise, single-word answers and poor quality audio and much shorter answers.	Cross-case analysis and data from these participants did not add to the depth of understanding.

Information Power	Remains low	Variation improved which does potentially increase information power	Remains low	Low from these participants but still high overall due to existing quality data collected.	Remains mixed – addition of these interviews did not change information power.
5 additional interviews recruited from posters and Footsteps Festival social media responses connected to local pain groups	Study aim remains broad	Further variation through experiences of people from more diverse sources than a consultant-based dental service with different experiences of the phenomenon in question	Not closely guided by theory	Additional quality dialogue and in-depth discussion.	Both increased coverage and depth generated from these interviews.
Information Power	14 interviews complete at this stage. Despite broad aims, convergence of data from interviews is starting to support increased information power.	Moderate information power through an increased range of interviews of people experiencing the phenomena. Underrepresentation of men and people of colour means there are gaps.	Increasing power through increased data collected.	High due to depth of discussions.	Becoming moderate due to the increasing breadth and depth of interview data.
Additional 5 interviews attempting to target people underrepresented	interviews complete. Increasing coverage of broad study objectives.	Increasing diversity of the sample. Two of these three were people of colour, both professional highly educated people.	Growing sample is adding data to support analysis despite lack of	High quality in-depth interviews.	Increased breadth and depth

in interviews to date (Through TMJA)		All female and men remain underrepresented. Different experiences have also been covered within the 17 interviews to date regarding type of TMD, co-morbidity, healthcare journey and impact of pain.	specific theoretical framework.		
Information Power	Moderate to High Information Power	Moderate to High Information Power.	Moderate to High Information power despite broader theoretical perspective	High information power	High Information power based on breadth and depth of data collected
